# Oral Health in Breast Cancer Women with Vitamin D Deficiency: A Machine Learning Study

**DOI:** 10.3390/jcm11164662

**Published:** 2022-08-09

**Authors:** Martina Ferrillo, Mario Migliario, Nicola Marotta, Lorenzo Lippi, Alessandro Antonelli, Dario Calafiore, Valerio Ammendolia, Leonzio Fortunato, Filippo Renò, Amerigo Giudice, Marco Invernizzi, Alessandro de Sire

**Affiliations:** 1 Dentistry Unit, Department of Health Sciences, University of Catanzaro “Magna Graecia”, 88100 Catanzaro, Italy; 2 Dentistry Unit, Department of Translational Medicine, University of Eastern Piedmont, 28100 Novara, Italy; 3 Physical Medicine and Rehabilitation Unit, Department of Medical and Surgical Sciences, University of Catanzaro “Magna Graecia”, 88100 Catanzaro, Italy; 4 Physical and Rehabilitative Medicine, Department of Health Sciences, University of Eastern Piedmont, 28100 Novara, Italy; 5 Translational Medicine, Dipartimento Attività Integrate Ricerca e Innovazione (DAIRI), Azienda Ospedaliera SS. Antonio e Biagio e Cesare Arrigo, 15121 Alessandria, Italy; 6 Physical Medicine and Rehabilitation Unit, Department of Neurosciences, ASST Carlo Poma, 46100 Mantova, Italy; 7 Innovative Research Laboratory for Wound Healing, Health Sciences Department, Università del Piemonte Orientale, 28100 Novara, Italy

**Keywords:** vitamin D, oral health, periodontal disease, breast cancer, osteoporosis, bone loss, cancer treatment-induced bone loss, rehabilitation

## Abstract

Breast cancer (BC) survivors treated with aromatase inhibitors (AIs) commonly show several pathological issues, including poor oral health, bone health impairment, and vitamin D deficiency. However, to date, oral health issues in BC survivors treated with AIs have been poorly investigated and their relationship with vitamin D deficiency are far from being understood. This study aimed to evaluate the correlation between oral health and vitamin D status in BC survivors undergoing treatment with AIs through a machine learning approach. In this cross-sectional study, we included post-menopausal BC women with vitamin D deficiency undergoing AIs therapy. The outcome measures were the following: oral health indexes as the Decayed, Missing, and Filled Permanent Teeth Index (DMFT); serum levels of 25(OH)D_3_; Bone Mineral Density (BMD); and the diagnosis of osteoporosis. We included 41 post-menopausal BC women, mean aged 66.10 ± 8.47 years, with mean serum levels of vitamin D of 14.63 ± 6.62 ng/mL. Furthermore, 56.10% of patients had a diagnosis of osteoporosis and 36.59% were osteopenic. DMFT was significantly related to smoking (*p*-value = 0.005) and dental floss use (*p*-value = 0.001). There was a significant correlation between DMFT and vitamin D levels (Pearson’s r: −0.73; *p*-value = 0.001). The regression machine learning model showed that vitamin D status and the use of dental floss were the most relevant variables in terms of correlation with DMFT. In conclusion, vitamin D deficiency, inadequate use of dental floss, and smoking had a negative impact on oral health in BC women. Thus, vitamin D deficiency screening and supplementation and a prompt oral rehabilitation plan should be suggested and implemented in the complex treatment framework of BC survivors undergoing treatment with AIs.

## 1. Introduction

Breast cancer (BC) is the most common cause of cancer-related deaths among women worldwide [1], albeit the incidence has declined during the last few decades [2,3]. Indeed, the death rate has dropped from its peak for female breast cancer by 40% [1], probably due to an adequate combination of early screening programs and improvement in adjuvant therapy, such as the aromatase inhibitors (AIs)—commonly used in estrogen-positive tumors to prevent recurrence [4]. These drugs aim to reduce the effects of estrogens on breast tissue [5,6], but could induce several adverse events, including a negative impact on bone health [7,8]. The aromatase enzyme is expressed not only in BC tissue but also in bone tissue [9], thus, AIs could lead to a lower bone mineral density (BMD) with a resulting increased risk of incident fragility fractures, leading to the well-known condition defined as cancer treatment-induced bone loss (CTIBL) [10,11]. Therefore, an adequate screening for bone health status in BC women undergoing AIs treatment should be recommended to define an appropriate anti-osteoporotic drug therapy, including oral bisphosphonates (BPs), zoledronic acid, or denosumab [12,13].

In this context, previous studies showed that BC survivors treated with AIs have worse subjective oral health, periodontal health, and oral health-related quality of life [14,15]. Hence, bisphosphonate-related osteonecrosis of the jaws [16], oral lesions [15], caries [17], and periodontal tissue diseases (e.g., gingivitis and periodontitis) [18,19] may occur in a higher percentage of BC women undergoing cancer treatments and anti-osteoporotic drugs. Similarly, vitamin D plays a key role in different fields of medicine, including dentistry, and acts through several mechanisms, including cellular proliferation and differentiation, cell maturation, and innate immune system response [20]. Moreover, high serum vitamin D3 levels may be strongly associated with an increase in the overall survival rate of BC patients [21,22], and this could be related to the fact that both vitamin D3 and vitamin D receptors (VDR) are involved in BC pathogenesis. Thus VDR, which is expressed at a systemic level, may act as a negative tumor suppressor regulator in physiological conditions and could be impaired in cancer patients, thereby promoting cancer transformation and other cancer-related sequelae, including bone health impairment [23,24].

In this context, previous studies suggested that vitamin D could promote the synthesis of antimicrobial peptides and inhibit antigen-induced T-cell proliferation as well as cytokine production (e.g., interleukin (IL)-2 and interferon (IFN)-γ), resulting in a relevant anti-inflammatory effect [25,26]. Furthermore, vitamin D supplementation seems to increase the serum concentration of anti-inflammatory cytokine IL-10 and decrease pro-inflammatory cytokines like tumor necrosis factor (TNF)-α [27,28,29]. In light of these anti-inflammatory and antimicrobial properties, several studies showed a correlation between low vitamin D serum levels and periodontal disease (PD) [29,30,31]. PD is one of the most common chronic inflammatory diseases affecting the tissues surrounding the teeth and is characterized by local tissue destruction initiated by the exposure to bacterial plaque and their metabolic bioproducts [32]. In vitro studies have shown that vitamin D3 might induce the expression of the antimicrobial peptide, LL-37, in cultured gingival epithelial cells, and that vitamin D supplementation leads to a reduction in the viability of the periodontal pathogen, Aggregatibacter actinomycetemcomitans, on the surface of the cells [33,34]. Moreover, the same in vitro studies suggested that LL-37, induced by vitamin D, exhibits antimicrobial activity against Porphyromonas gingivalis and other pathogens associated with PD pathogenesis [33,34]. In this context, Freudenheim et al. [35] demonstrated that PD might significantly increase BC risk and suggested a possible role of the oral microbiome in BC pathogenesis and prevention.

**Figure 1:**
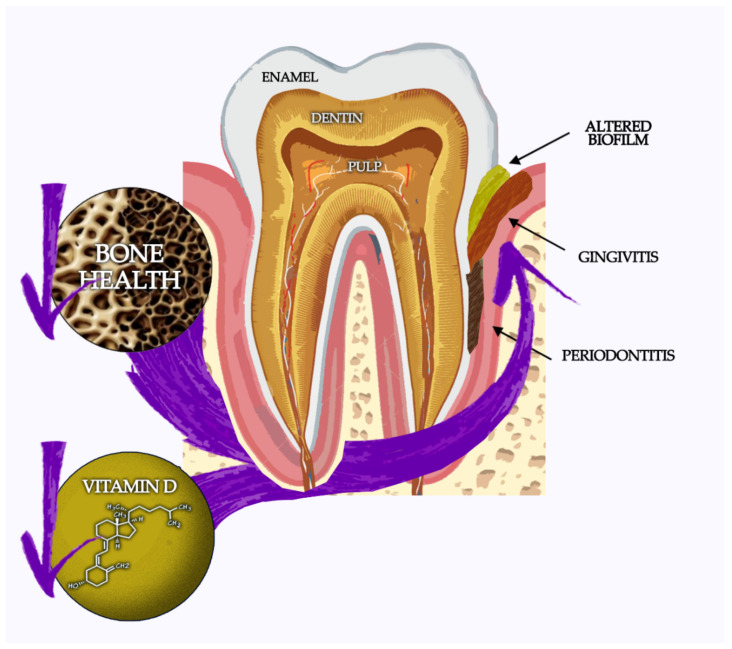
Correlation among oral health, bone mineral density, and vitamin D status.

Albeit the negative influence on quality of life in BC survivors [36], chronic oral diseases are frequently neglected issues in these women and, considering the higher incidence of anti-osteoporotic drugs-related oral sequelae in cancer patients [37], a preventive oral health evaluation should be performed before starting pharmacological CTIBL treatment (see Figure 1).

The correlation among oral health, bone health, and vitamin D status has been previously investigated in different conditions [29,30,31]. However, to date, the pathophysiological mechanisms underpinning this linkage in BC survivors has not been widely investigated yet. In light of these considerations, this study aimed to evaluate the correlation between oral hygiene and vitamin D status in BC survivors treated with AIs through a machine learning approach.

## 2. Materials and Methods

### 2.1. Study Participants

In this observational cross-sectional study, we included BC women over a 12-month period (April 2021–March 2022) that were referred to the Dentistry Unit of the University Hospital “Maggiore della Carità” of Novara, Italy. The inclusion criteria were the following: (i) BC women in post-menopausal status; (ii) vitamin D deficiency ([25(OHvit.D] < 30 ng(mL); (iii) ongoing AIs therapy; and (iv) understanding and signing the informed consent. The exclusion criteria were the following ones: (i) age < 50 years old; (ii) previous fragility fractures; (iii) treatment with corticosteroids, immunoglobulin, or immunosuppressive drugs; (iv) major concurrent diseases; (v) fully edentulous patients; and (vi) diagnosis of COVID-19.

The study was approved by the Local Ethic Committee (CE n. 392-61/10) and respected the Declaration of Helsinki, with pertinent National and International regulatory requirements. All participants were asked to carefully read and sign an informed consent, taking precautions to protect the privacy of patients. Lastly, the study was performed in accordance with the “Strengthening the Reporting of Observational Studies in Epidemiology” (STROBE) Guidelines for cross-sectional studies.

### 2.2. Outcome Measures

The following demographic and anamnestic data were collected: age, body mass index (BMI), smoking habit, BC grading, radiotherapy, hormone therapy, chemotherapy, breast cancer related-lymphedema; axillary web syndrome, Human Epidermal Growth Factor Receptor 2 (HER2) positivity, and percentage of Ki-67 protein (Ki-67).

The following data regarding bone health were also assessed: serum levels of 25(OH)D3 (ng/mL), serum calcium (mg/dL), serum parathyroid hormone (PTH) (pg/mL), lumbar spine (LS) bone mineral density (BMD), LS Tscore, LS Zscore, femoral neck (FN) BMD, FN Tscore, FN Zscore (through the dual X-ray absorptiometry, DXA), diagnosis of osteoporosis, and diagnosis of osteopenia.

Furthermore, all the participants underwent an oral health specialist evaluation in order to assess the following outcome measures: the Decayed, Missing, and Filled Permanent Teeth Index (DMFT) [38], to assess dental caries prevalence as well as dental treatment needs; the Oral Hygiene Index (OHI) [39], for the presence of debris/stain and calculus on the dental elements; the Plaque Control and Record Index (PCR) [40], to assess the presence of plaque on the dental elements; the Periodontal Screening and Recording Index (PSR), to assess periodontal status; the Winkel Tongue Coating Index (WTCI) [41], to evaluate the amount of tongue coating.

### 2.3. Statistical Analysis

A statistical analysis was performed using R (v3.5.2 R Core Team, Vienna, Austria). The continuous variables are presented as means ± standard deviations, and the categorical variables are expressed as counts (percentages). The Shapiro–Wilk test was performed to assess the distribution of all continuous data. We related all the dichotomous variables to the DMFT through a logistic regression analysis. Regarding continuous data, Pearson’s correlation coefficients and regression analyses assessed associations and correlations regarding both bone and oral health status and clinical and demographic features of the study participants. A cut-off *p*-value of 0.05 was considered statistically significant.

Furthermore, a random forest regression model, as a machine learning approach, was conducted to estimate the importance of variables based on how best or worse the prediction would be if one or more variables are removed, thereby weighting the elimination of predictor variables. In this context, the Gini Variable Importance estimates the importance of individual predictors via the changes in each node impurities at each split in each tree of the random forest. This Gini importance or mean decrease in the impurity of the node is the difference between impurities of a node, with or without a variable in the model, and so the weighted sum of the impurities in the two descendent nodes. Thus, we analyzed dichotomous variables as a measure of the importance of characteristics in the random forest, with there being an inherent imbalance towards continuous variables, or ordinal variables with multiple categories, so we dichotomized vitamin D using 20 ng/mL as a cut-off.

## 3. Results

Out of 46 subjects, 5 did not match the inclusion/exclusion criteria and were excluded; thus, 41 post-menopausal BC women (mean aged 66.1 ± 8.47 years) were included in the final analysis. Demographic and clinical characteristics of the patients enrolled are summarized in Table 1.

The cohort showed a mean serum level of 25(OH)vitamin D of 14.63 ± 6.62 (ng/mL), a mean LS BMD of 0.92 ± 0.19 g/cm^2^, and a FN BMD of 0.74 ± 0.1 g/cm^2^. Moreover, 56.10% of the patients included in the study had osteoporosis and 36.59% had osteopenia. For further details about bone health, see Table 2.

Oral hygiene status was reported in Table 3.

Two-thirds of the participants reported use a manual toothbrush, less than half used dental floss, and only a quarter used the tooth cleaner. The DMFT index had an average value of 17.44 ± 6.76. Regarding the PSR index assessment, only 4 patients (9.76%) had normal periodontal health status, 10 (24.39%) had gingivitis, 22 (53.66%) had moderate periodontitis, and 5 (12.2) had severe periodontitis.

Therefore, we performed a correlation analysis that relates hither DMFT to the dichotomous variables for a logistic regression. The increase in the DMFT is related to smoking (*p*-value = 0.005), whereas a decrease in the score is significantly related to the use of the dental floss (*p*-value = 0.001). The results of the logistic regression between the DMFT, oral health habits, and BC characteristics are shown in Table 4.

Lastly, we performed a correlation for the continuous anthropometric, oral, and bone health variables with the DMFT. We demonstrated a statistically significant correlation between DMFT and 25(OH)vitamin D serum levels with a Pearson’s r of −0.73 (*p*-value = 0.001). For further details, see Table 5.

To weigh the influence of each variable on the DMFT score, we performed a Random Forest Regression Model, generating 66 trees, with a test Mean Square Error of 0.88, an Out-of-Bag Error of 0.74, and, lastly, an R^2^ = 0.716. After dichotomizing the vitamin D values as less than or greater than 20, we have ranked the variables according to the mean decrease in accuracy and increase in node purity at each split of random forest generation. Therefore, we reported through a regression machine learning model that the following most important variables are vitamin D serum levels and the use of dental floss.

## 4. Discussion

Oral health in BC survivors is often a neglected issue with relevant implications in terms of disability and poor quality of life. This point could have relevant clinical implications considering that most BC survivors are treated with AIs and should undergo a pharmacological anti-resorptive treatment. These drugs may have a negative impact on oral health in the general cancer population; therefore, these patients should be screened for oral health pathological conditions and eventually treated to prevent the occurrence of even worse disabling sequelae. In this scenario, this cross-sectional study aimed to assess the correlation among bone health, vitamin D deficiency, and oral hygiene, and showed through a machine learning approach a possible correlation among bone and oral health with vitamin D deficiency in BC women undergoing AIs.

Our findings highlighted a high prevalence of osteoporosis (56.10%) and osteopenia (36.59%), and a prevalence of periodontitis of 65.8%, which is significantly higher than the general population [42]. These data are in line with a recent meta-analysis showing that post-menopausal women with osteoporosis or osteopenia exhibit greater loss of periodontal attachment compared with women with normal bone mineral density [43]. The authors suggested that periodontal disease might significantly increase the risk of BC by 1.22-fold [43], which is likely due to the immune response to oral bacterial flora that might increase systemic inflammation and oxidative stress with negative implications in the tumorigenesis process [44].

On the other hand, a significant correlation between DMFT and smoking subjects (*p*-value = 0.005) has been shown in accordance with previous studies, highlighting the detrimental consequences of smoking on oral hygiene [45,46]. However, it should be noted that smoking could also have a negative impact on BMD. Furthermore, the most recent joint position statement of interdisciplinary cancer and bone societies integrated smoking in the risk factor algorithm to tailor pharmacological management of CTIBL in patients receiving AIs [47].

Taken together, our results emphasized the role of a healthy lifestyle behavior, including smoking cessation in the comprehensive management of BC patients. These results suggested intriguing implications in a comprehensive counseling approach, thereby improving awareness of BC patients about the importance of lifestyle medicine in overall well-being of patients with cancer and promoting not only better oral health, but also bone health.

Furthermore, dental floss has been associated with DMFT scores (*p*-value = 0.001), suggesting that good oral hygiene practices (e.g., using dental floss) should definitely be implemented in the management of BC patients. Indeed, oral hygiene plays a crucial role in BC women receiving AIs, since growing evidence underlines the need for anti-resorptive drugs preventing CTIBL.

Intriguingly, a regression machine learning model confirmed the strict correlation between DMFT scores with the use of dental floss and smoking habits. In recent years, a growing interest has been rising in machine learning solutions, considering that these statistical approaches might have a crucial role in developing self-improving technological models. To better characterize the role of different variables in BC patients, machine learning-based statistical models have already been successfully integrated into the most recent approaches for oncologic patients [48,49,50]. In this scenario, novel multivariant statistical methods might improve correlation assessments by including several groups of continuous variables and clustering the study participants through a machine learning model [51,52].

Interestingly, our regression machine learning model analysis highlighted that vitamin D serum levels have been associated with higher weight of influence on the DMFT score. In the last few decades, the nutritional consequences of vitamin D deficiency on periodontal health represented a matter of interest [20,53,54,55]. By a recent systematic review with meta-analysis, Machado et al. [53] reported that patients with chronic periodontitis had lower serum levels of vitamin D than periodontally healthy patients. The exact mechanics by which vitamin D may influence periodontal status is still unknown; however, Gao et al. [54] recently showed that the human cationic antimicrobial protein of 18 kDa (hCAP-18)/LL-37 expression might be induced by vitamin D and Porphyromonas gingivalis-LPS, supporting that the vitamin D pathway is likely to exist in both human gingival fibroblasts and periodontal ligament cells and might play a role in the immune defense of periodontal soft tissues.

On the other hand, vitamin D has widely documented effects on bone health management, playing a key role in both non-pharmacological and pharmacological osteoporosis interventions [56]. In this context, osteoporosis is a systemic condition affecting the whole skeletal system with potential implications for bone structures of the jawbones and alveolar bone in patients, such as cancer survivors at higher risk of osteonecrosis of the jaw [57]. In addition, the previously reported close link between oral health status and osteoporosis might be partly explained by systemic inflammation that characterizes both conditions. In this scenario, the effects of vitamin D on immune systems are widely accepted, and several reports underline the role of vitamin D in inflammation downregulation. In particular, vitamin D might promote the upregulation of MAP kinases and inhibit the NF-kB signaling pathway, with crucial implications for cytokine serum levels, the prostaglandin inflammation pathway, and immune cells [58,59]. Moreover, recent studies highlighted that B cells, CD4+ and CD8+ T cells, dendritic cells, and macrophages express VDR and might be selectively targeted by vitamin D [60,61]. In this scenario, the effects of vitamin D in systemic inflammation is currently considered an important hallmark in BC patients [62]. Therefore, vitamin D’s positive effects in immune regulation might have not only a positive role in oral health management and bone health, but also in the downregulation of inflammatory mediators that promote tumor growth or risk of recurrence [61,62]. Concurrently, periodontitis and gingivitis have been related to increases in immune response and systemic inflammation [63,64].

Therefore, our findings might support the need for vitamin D supplementation in BC patients, not only to improve bone health and to prevent CITBL, but also to reduce periodontitis and gingivitis risks, with crucial implications on systemic inflammation and patients’ HRQoL.

In line with these considerations, oral hygiene screening should be effectively integrated in the comprehensive management of BC survivors, as is already performed for patients with disabling neurological conditions who need prompt oral rehabilitation [65,66]. Furthermore, a specific assessment of BC survivors at high risk of oral health issues should include patients with hypovitaminosis D, smokers, and those not using dental floss. This screening might have a relevant role in reducing sanitary costs and focusing health care resources for a patient-tailored oral rehabilitation intervention.

However, we are aware that this study is not free from limitations. First, we had a small sample, probably due to the monocentric study design and to the strict eligibility criteria. Second, there is a lack of data on the subcomponents of the DMFT index and on other potential confounding factors (e.g., nutritional status, physical activity, etc.) that might influence the outcomes. Lastly, we did not provide data on the panoramic X-ray images of these patients, albeit it could have been interesting to assess the BMD also using the radio-morphometric indices of the mandible [67,68,69] and comparing these findings with the DXA examinations.

## 5. Conclusions

Taken together, the results of this cross-sectional study underlined a relevant correlation between vitamin D serum levels and oral health in BC women treated with AIs through a regression machine learning model. Furthermore, vitamin D deficiency, inadequate use of dental floss, and smoking had a negative impact on oral health in a cohort of post-menopausal BC women. In this scenario, we could recommend that the comprehensive screening and treatment framework known as “oral rehabilitation” should be included in the complex multidisciplinary management of BC survivors to reduce disabling sequalae occurrence and improve the HRQoL of these women. This could have relevant clinical implications and might pave the way to the development of a self-improving machine learning algorithm to better address the need for a tailored and multidisciplinary management model of BC survivors.

## Figures and Tables

**Table 1:** Study population characteristics (*n* = 41).

Mean age (years)	66.1 ± 8.47
BMI (kg/m^2^)	24.83 ± 4.41
Smokers (*n*, %)	12 (29.27%)
Grading	
G1 (*n*, %)	5 (12.20%)
G2 (*n*, %)	26 (63.41%)
G3 (*n*, %)	10 (24.39%)
HER2 (*n*, %)	9 (21.95%)
Ki-67	
*-Low (<18%) (n, %)*	24 (58.54%)
*-High (>18%) (n, %)*	7 (17.07%)
RT (*n*, %)	31 (75.61%)
HT (*n*, %)	41 (100.00%)
CT (*n*, %)	17 (41.46%)
BCRL (*n*, %)	9 (21.95%)
AWS (*n*, %)	13 (31.71%)

Continuous variables are expressed as means ± standard deviations; categorical variables are expressed as counts (percentages). Abbreviations: HER2/neu: Human Epidermal Growth Factor Receptor 2; RT: radiotherapy; HT: hormone therapy; CT: chemotherapy; BCRL: Breast Cancer related-lymphedema; AWS: Axillary web syndrome.

**Table 2:** Vitamin D status and bone health outcomes in the study population (*n* = 41).

Serum 25OH-Vit.D (ng/mL)	14.63 ± 6.62
Serum 25(OH)vit. D ≥ 20 and <30 ng/mL (*n*, %)	10 (24.39%)
Serum 25(OH)vit. D ≥ 10 and <20 ng/mL (*n*, %)	21 (51.22%)
Serum 25(OH)vit. D < 10 ng/mL (*n*, %)	10 (24.39%)
Serum calcium (mg/dL)	9.23 ± 0.54
Serum PTH (pg/mL)	43.5 ± 12.24
LS BMD (g/cm^2^)	0.92 ± 0.19
LS Tscore	−1.91 ± 1.31
LS Zscore	−0.45 ± 1.29
FN BMD (g/cm^2^)	0.74 ± 0.1
FN Tscore	−1.94 ± 0.89
FN Zscore	−0.54 ± 0.83
Osteoporosis (*n*, %)	23 (56.10%)
Osteopenia (*n*, %)	15 (36.59%)

Continuous variables are expressed as means ± standard deviations; categorical variables are expressed as counts (percentages). Abbreviations: 25(OH)vit. D: 25-hydroxy-vitamin D; PTH: parathyroid hormone; LS: lumbar spine; BMD: bone mineral density; FN: femoral neck.

**Table 3:** Oral hygiene status in the study population (*n* = 41).

Manual toothbrush (*n*, %)	28 (68.29%)
Electric toothbrush (*n*, %)	13 (31.71%)
Dental floss (*n*, %)	16 (39.02%)
DMFT	17.44 ± 6.76
OHI	
*≤1.2* (*n*, %)	*9 (21.95%)*
*>1.2 ≤ 3.0* (*n*, %)	*14 (34.15%)*
*>3.0≤ 6.0* (*n*, %)	*18 (43.90%)*
PCR	
*From 0% to 25%* (*n*, %)	*10 (24.39%)*
*>25% and ≤50%* (*n*, %)	*12 (29.27%)*
*>50% and ≤75%* (*n*, %)	*12 (29.27%)*
*>75%* (*n*, %)	*7 (17.07%)*
PSR	
*Normal periodontal health status* (*n*, %)	*4 (9.76%)*
*Gingivitis* (*n*, %)	*10 (24.39%)*
*Mild/moderate periodontitis* (*n*, %)	*22 (53.65%)*
*Severe periodontitis* (*n*, %)	*5 (12.2%)*
WTCI	
*Grade 0* (*n*, %)	*5 (12.2%)*
*Grade 1* (*n*, %)	*21 (51.22%)*
*Grade 2* (*n*, %)	*15 (36.58%)*

Continuous variables are expressed as means ± standard deviations; categorical data are expressed as counts (%). Abbreviations: DMFT = Decayed, Missing and Filled Permanent Teeth; OHI = Oral Hygiene Index; PCR = Plaque Control and Record; PSR = Periodontal Screening and Recording; WTCI = Winkel Tongue Coating Index.

**Table 4:** Univariate Logistic regression between the Decayed, Missing, and Filled Permanent Teeth Index (DMFT), oral hygiene habits, and breast cancer characteristics.

Variable	Odd Ratio (95% CI)	*p*-Value
Smoke	1.17 (1.03–1.32)	0.005 *
Manual toothbrush use	0.83 (0.73–1.11)	0.052
Electric toothbrush use	0.84 (0.75–1.09)	0.052
Dental floss use	0.83 (0.73–0.95)	0.001 *
HER2	1.05 (0.94–1.18)	0.522
Ki-67	1.01 (0.92–1.10)	0.643
RT	0.96 (0.86–1.07)	0.651
HT	1.06 (0.94–1.19)	0.532
CT	0.97 (0.88–1.06)	0.522
BCRL	1.01 (0.91–1.13)	0.573
AWS	1.06 (0.96–1.17)	0.647

Abbreviations: CI: confidence interval; HER2: Human Epidermal Growth Factor Receptor 2; RT: radiotherapy; HT: hormone therapy; CT: chemotherapy; AWS: Axillary web syndrome; BCRL: Breast Cancer-Related Lymphedema; * = *p* value <0.05.

**Table 5:** Variable importance in regression random forest model in terms of correlation with Decayed, Missing, and Filled Permanent Teeth Index (DMFT).

	Mean Decrease in Accuracy	Total Increase in Node Purity
25OH-Vit.D serum levels	0.22	1.46
Dental floss	0.08	1.36
Smoke	0.11	0.99
HER2	0.06	0.50
BCRL	0.00	0.39
CT	0.00	0.34
Toothbrush	−0.02	0.30
Ki-67	−0.01	0.27
AWS	0.00	0.21
RT	−0.01	0.16
HT	0.00	0.00

Abbreviations: 25(OH)vit. D: 25-hydroxy-vitamin D; HER2: Human Epidermal Growth Factor Receptor 2; BCRL: Breast Cancer-Related Lymphedema; CT: chemotherapy; AWS: Axillary web syndrome; RT: radiotherapy; HT: hormone therapy.

## Data Availability

Dataset is available on request.
